# Toward Personalized NSAID Therapy in Osteoarthritis: The Right Patient, the Right Treatment, at the Right Time

**DOI:** 10.3390/life16081303

**Published:** 2026-08-08

**Authors:** Valerica Creanga Zarnescu, Liliana Mititelu-Tartau, Ilie Onu, Daniel Andrei Iordan, Liliana-Lăcrămioara Pavel

**Affiliations:** 1 The School for Doctoral Studies in Biomedical Sciences, “Dunărea de Jos” University of Galați, 800008 Galați, Romania; valerica.creanga-zarnescu@ugal.ro; 2 Center of Physical Therapy, Rehabilitation and Wellness, “Dunărea de Jos” University of Galati, 800008 Galati, Romania; 3 Grigore T. Popa University of Medicine and Pharmacy Iasi, 700454 Iasi, Romania; liliana.tartau@umfiasi.ro; 4 Department of Individual Sports and Kinetotherapy, Faculty of Physical Education and Sport, Dunarea de Jos University of Galati, 800008 Galati, Romania; 5 Medical Department, Faculty of Medicine and Pharmacy, “Dunarea de Jos“ University of Galati, 800010 Galati, Romania; liliana.pavel@ugal.ro

**Keywords:** osteoarthritis, non-steroidal anti-inflammatory drugs, pain phenotyping, nociceptive pain, nociplastic pain, personalized medicine, treatment response, risk stratification, clinical decision-making

## Abstract

Background: Non-steroidal anti-inflammatory drugs (NSAIDs) remain the cornerstone of pharmacological treatment for osteoarthritis (OA) and are recommended by international guidelines for the management of symptomatic pain. Despite their well-established efficacy, substantial interindividual variability exists in treatment response, with some patients experiencing meaningful pain relief while others derive little clinical benefit despite appropriate drug selection and dosing. This variability reflects, at least in part, the biological heterogeneity of OA pain. Objective: To review the mechanisms underlying variability in NSAID responsiveness in OA and to propose a practical framework for personalized NSAID prescribing based on pain phenotype, individual safety profile, and treatment timing. Methods: A narrative review of the contemporary scientific literature was conducted using major biomedical databases to summarize the current evidence on phenotype-guided NSAID therapy in OA. Particular attention was given to the emerging concepts of nociceptive, nociplastic, and neuropathic-like pain phenotypes and their implications for personalized anti-inflammatory therapy. Results: Current evidence indicates that OA pain is a heterogeneous and dynamic condition in which inflammatory nociceptive, nociplastic, and neuropathic-like mechanisms coexist to varying degrees. NSAIDs primarily target inflammatory nociceptive pain by inhibiting cyclooxygenase (COX)-mediated prostaglandin synthesis and reducing peripheral sensitization. Consequently, patients with predominantly inflammatory nociceptive pain are the most likely to benefit from NSAID therapy, whereas those with predominant nociplastic or neuropathic-like pain mechanisms may require alternative or multimodal treatment strategies. Beyond pain phenotype, optimal NSAID selection should integrate cardiovascular, gastrointestinal, and renal risk assessment, recognizing the important pharmacological and safety differences among individual agents. Treatment timing is also clinically relevant, as anti-inflammatory therapy appears most effective when initiated during periods of active inflammatory nociceptive pain. Based on the available evidence, we propose a conceptual clinical decision framework integrating patient selection, NSAID choice, and treatment timing. Conclusions: Personalized NSAID prescribing should move beyond a diagnosis-based approach toward a mechanism-based strategy that integrates pain phenotyping, individualized safety assessment, and appropriate treatment timing. The proposed framework is built upon three complementary principles, identifying the right patient, selecting the right treatment, and initiating therapy at the right time. It provides a practical foundation for implementing precision medicine in the pharmacological management of OA.

## 1. Introduction

Osteoarthritis (OA) is the most common form of arthritis worldwide and one of the leading causes of pain and disability among older adults [1]. The principal risk factors for OA include advancing age, female sex, obesity, and adverse mechanical factors [2]. The knee, hip, and hand are the most commonly affected peripheral joints, and patients typically experience persistent joint pain, stiffness, reduced mobility, and progressive functional limitation, leading to impaired quality of life and a substantial socioeconomic burden.

Pain is the predominant symptom driving healthcare utilization and therapeutic decision-making in OA. Consequently, current management strategies focus on pain relief and functional improvement through a combination of non-pharmacological and pharmacological interventions. Among the available pharmacological options, NSAIDs remain the cornerstone of symptomatic treatment and are consistently recommended by international guidelines for the management of OA-related pain [3].

The rationale for NSAID use is based on their ability to interfere with key inflammatory pathways involved in peripheral pain sensitization. Osteoarthritic joints are characterized by the production of inflammatory mediators, including prostaglandins (PGs), cytokines, and nerve growth factor (NGF), which reduce the activation threshold of peripheral nociceptors and amplify pain transmission. By inhibiting cyclooxygenase (COX) enzymes, NSAIDs decrease prostaglandin synthesis, thereby attenuating inflammatory signaling and reducing peripheral nociceptor sensitization [4]. In addition to their well-established peripheral anti-inflammatory effects, experimental evidence suggests that some NSAIDs may also exert central analgesic effects by reducing prostaglandin production within the central nervous system (CNS), potentially influencing spinal pain processing and central sensitization.

The efficacy of NSAIDs in OA has been consistently demonstrated in randomized controlled trials (RCTs) and meta-analyses [5,6,7]. Among these, the network meta-analysis by da Costa et al., which included 74 RCTs involving 58,556 patients, identified diclofenac 150 mg/day and etoricoxib 60 mg/day as the treatments with the highest probability of achieving clinically meaningful pain reduction compared with placebo. Diclofenac 150 mg/day was also the only intervention consistently associated with clinically relevant improvements in physical function. Furthermore, significant dose–response relationships were observed for celecoxib, diclofenac, and naproxen, emphasizing the importance of adequate dosing when prescribing anti-inflammatory therapy [6].

Despite this robust evidence, the clinical response to NSAID therapy varies considerably among patients. While some individuals experience substantial pain relief and meaningful functional improvement, others derive little or no benefit despite receiving the same drug at an appropriate dose and duration [3,4]. Traditionally, this variability has been attributed to differences in pharmacokinetics, disease severity, comorbidities, treatment adherence, or duration of therapy. However, accumulating evidence suggests that the biological heterogeneity of OA pain itself may represent a major determinant of treatment responsiveness.

At the same time, the clinical use of NSAIDs is limited by potentially serious adverse effects, with cardiovascular (CV), gastrointestinal (GI), and renal complications representing the principal concerns associated with both short- and long-term therapy. Consequently, current prescribing strategies primarily emphasize balancing analgesic efficacy against individual safety risks [7,8,9]. Although this approach is essential for minimizing adverse events, it provides limited guidance regarding the likelihood of therapeutic success. An equally important clinical question, therefore, remains largely unanswered: why do some patients experience substantial benefit from NSAID therapy whereas others obtain little or no improvement despite receiving the same treatment?

Increasing evidence indicates that OA pain is not a homogeneous entity but rather a multidimensional condition involving varying contributions of nociceptive, neuropathic-like, and nociplastic mechanisms. Accordingly, patients with similar radiographic disease severity may exhibit distinct pain phenotypes and markedly different probabilities of responding to anti-inflammatory treatment [10]. Because NSAIDs primarily target inflammatory nociceptive mechanisms through inhibition of prostaglandin synthesis, their effectiveness is likely to depend not only on the specific drug selected but also on identifying the appropriate patient and initiating treatment while inflammatory mechanisms remain major contributors to symptom generation [1,5].

These observations support a shift from the traditional one-size-fits-all approach toward a personalized strategy for NSAID prescribing based on three complementary principles: selecting the right patient, choosing the right treatment, and initiating therapy at the right time. Integrating pain phenotyping with conventional CV, GI, and renal risk assessment may improve patient selection, optimize treatment response, and minimize unnecessary drug exposure and treatment-related adverse events.

Therefore, this narrative review aims to summarize current evidence regarding the mechanisms underlying variability in NSAID responsiveness in OA and to propose a practical clinical framework for personalized NSAID prescribing based on pain phenotype, individual safety assessment, and treatment timing. Furthermore, we present a clinically applicable decision-making algorithm designed to assist healthcare professionals in selecting the right NSAID for the right patient at the right time.

To our knowledge, most published reviews have focused primarily on the efficacy or safety of NSAIDs. In contrast, this review integrates current evidence on pain phenotyping, individualized risk stratification, and treatment timing into a practical framework for personalized NSAID prescribing.

Although accumulating mechanistic, translational, and clinical evidence suggests that patients with predominantly inflammatory nociceptive pain may derive greater benefit from NSAID therapy than those with nociplastic or neuropathic-like pain characteristics, direct evidence from prospective phenotype-stratified RCT remains limited. Therefore, the framework proposed in this review should be interpreted as a conceptual, evidence-informed approach intended to support clinical reasoning rather than as a validated clinical decision tool.

## 2. Methods

This narrative review was conducted to summarize and critically appraise the current evidence regarding personalized NSAID therapy in OA, with particular emphasis on pain phenotyping, patient selection, treatment safety, and treatment timing. The review was not designed as a systematic review and therefore no formal protocol was registered.

A literature search was performed using the PubMed/MEDLINE, Scopus, and Google Scholar databases to identify relevant articles published from 2000 to 2026. To ensure the inclusion of landmark publications, several earlier studies considered fundamental to the understanding of NSAID pharmacology and osteoarthritis pain mechanisms were also included.

The search strategy combined Medical Subject Headings (MeSH) terms and free-text keywords, *including osteoarthritis, non-steroidal anti-inflammatory drugs, NSAIDs, pain phenotype, nociceptive pain, nociplastic pain, neuropathic pain, cyclooxygenase, cardiovascular risk, gastrointestinal risk, renal risk, precision medicine, personalized medicine, and clinical guidelines*. Additional relevant publications were identified through manual screening of the reference lists of selected articles.

Priority was given to high-quality evidence, including international clinical practice guidelines, systematic reviews, meta-analyses, RCTs, large observational studies, and key pharmacological reviews. Narrative reviews were considered when they provided clinically relevant background information or supported the interpretation of current evidence.

Only articles published in English were considered. Conference abstracts, editorials, letters to the editor, and studies lacking sufficient methodological detail were not included unless they provided unique or historically relevant information. The literature search was updated immediately before manuscript submission to ensure that the review reflected the most recent evidence available.

## 3. Why Do NSAIDs Work?

The analgesic efficacy of NSAIDs in OA is primarily explained by their ability to interrupt inflammatory pathways that drive peripheral nociceptive pain. Tissue injury and low-grade synovial inflammation characteristic of OA stimulate the release of arachidonic acid metabolites, cytokines, and growth factors, leading to increased production of PGs. These mediators lower the activation threshold of peripheral nociceptors, promote peripheral sensitization, and amplify pain transmission from affected joints. By reducing prostaglandin synthesis, NSAIDs directly target one of the key biological mechanisms responsible for inflammatory nociceptive pain [1].

The therapeutic effects of NSAIDs are mediated through inhibition of COX enzymes, which catalyze the conversion of arachidonic acid into PG2. Two major COX isoforms have been identified. COX-1 is constitutively expressed in most tissues and plays an essential role in physiological processes, including gastric mucosal protection, platelet aggregation, and maintenance of renal perfusion. In contrast, COX-2 is predominantly an inducible enzyme that is upregulated during inflammation and is largely responsible for the increased production of PGs associated with pain and inflammatory responses [11].

Individual NSAIDs differ considerably in their relative affinity for COX-1 and COX-2, resulting in important differences in both therapeutic efficacy and safety profiles [12]. Reported COX-1/COX-2 inhibitory ratios are derived from in vitro IC50 assays and should be interpreted with caution, as the reported values vary depending on the experimental system, assay conditions, and methodology used. Accordingly, the values presented below are intended to illustrate the relative pharmacological profiles of commonly used NSAIDs rather than provide absolute measures of enzyme selectivity. Traditional NSAIDs, including ibuprofen, naproxen, and indomethacin, inhibit both COX isoforms to varying degrees. Ibuprofen (COX-1/COX-2 IC50 ratio 0.5) and naproxen (0.7) demonstrate greater relative affinity for COX-1, whereas indomethacin exhibits a more balanced inhibitory profile (1.9). In contrast, meloxicam (18), diclofenac (29), and celecoxib (30) display increasing selectivity toward COX-2, while rofecoxib, which was subsequently withdrawn because of cardiovascular safety concerns, demonstrated markedly greater COX-2 selectivity (267) [13].

These pharmacological differences have important clinical implications. Inhibition of COX-1 contributes substantially to GI toxicity and impaired platelet function, whereas selective COX-2 inhibition was developed to preserve GI protection while maintaining anti-inflammatory efficacy [14]. However, increasing COX-2 selectivity has also been associated with a higher risk of CV events in susceptible individuals. Consequently, the selection of an NSAID should consider not only analgesic efficacy but also each patient’s CV, GI, and renal risk profile [15].

Although inhibition of prostaglandin synthesis represents the principal mechanism of action, additional pharmacological effects may also contribute to the clinical activity of certain NSAIDs. Experimental studies have shown that some agents can modulate neutrophil activation, suppress inducible nitric oxide synthase expression, reduce nitric oxide production, and influence neuroinflammatory pathways involved in both peripheral and central sensitization. While the clinical relevance of these supplementary mechanisms remains incompletely understood, they may partly explain differences in treatment response observed among individual NSAIDs and patient populations [16,17].

Importantly, the biological effects of NSAIDs also explain why their clinical efficacy varies among patients with OA. Because these drugs primarily interrupt PG-mediated inflammatory nociceptive signaling, they are expected to provide the greatest benefit when pain is predominantly driven by active peripheral inflammation. Conversely, patients in whom nociplastic or neuropathic-like mechanisms constitute the major contributors to pain are less likely to achieve meaningful analgesia from anti-inflammatory therapy alone. Thus, the effectiveness of NSAID treatment depends not only on the pharmacological properties of the selected drug but also on whether inflammatory nociceptive mechanisms remain the dominant drivers of an individual patient’s pain. This concept provides the biological rationale for a personalized approach to NSAID therapy in OA.

## 4. Why Do Some Patients Respond to NSAIDs While Others Do Not?

Although NSAIDs are recommended as first-line pharmacological therapy for symptomatic OA, treatment response varies considerably among patients. While some individuals experience substantial pain relief and functional improvement, others derive little or no benefit despite receiving the same drug at an appropriate dose. This variability cannot be explained solely by pharmacokinetic differences, disease severity, or treatment adherence. Increasing evidence suggests that it largely reflects the biological heterogeneity of OA pain itself [1].

Pain is currently defined by the International Association for the Study of Pain (IASP) as “an unpleasant sensory and emotional experience associated with, or resembling that associated with, actual or potential tissue damage” [10]. This definition recognizes pain as a multidimensional experience shaped by biological, psychological, and social factors rather than a simple consequence of structural tissue injury. Consistent with this concept, pain severity in OA frequently correlates poorly with radiographic disease severity, indicating that mechanisms beyond joint structural damage contribute substantially to symptom generation [10].

The transition from acute to chronic pain results from complex interactions among persistent peripheral nociceptive input, neuroplastic changes within the CNS, neuroimmune signaling, and genetic and epigenetic influences [18,19,20,21]. In OA, mechanical loading, cartilage degradation products, inflammatory mediators, cytokines, chemokines, and growth factors activate nociceptors located within the synovium, subchondral bone, ligaments, periosteum, and joint capsule. Sustained nociceptive input promotes peripheral sensitization, lowers activation thresholds, and increases pain responsiveness. Continued stimulation may subsequently induce central sensitization, whereby alterations in spinal and supraspinal pain processing amplify pain independently of ongoing peripheral inflammation [22,23,24].

These mechanisms support the current understanding that OA pain is not a single clinical entity but rather a heterogeneous condition comprising three major, frequently overlapping pain phenotypes: nociceptive, nociplastic, and neuropathic-like pain. Because NSAIDs primarily inhibit prostaglandin-mediated inflammatory nociceptive signaling, their clinical effectiveness depends largely on which pain mechanism predominates in an individual patient.

### 4.1. Nociceptive Pain: The Phenotype Most Likely to Respond to NSAIDs

Nociceptive pain results from actual or threatened tissue injury and remains the predominant pain phenotype in OA [6]. Traditionally, OA pain was considered almost exclusively nociceptive, reflecting local tissue damage and inflammation within the affected joint [25]. Current evidence demonstrates that inflammatory mediators, including PGs, cytokines, NGF, and matrix-degrading enzymes, play a central role in peripheral sensitization by lowering nociceptor activation thresholds and amplifying pain transmission [26,27,28,29].

Macrophages and other innate immune cells further contribute to this process through the release of inflammatory mediators and bidirectional communication with sensory neurons [30,31]. In addition, neuroinflammatory pathways involving Toll-like receptors, ADAMTS5 signaling, and NGF reinforce the close interaction between inflammation and nociception in OA [32,33].

Because NSAIDs directly inhibit prostaglandin synthesis and reduce peripheral inflammatory sensitization, patients whose symptoms are predominantly driven by inflammatory nociceptive mechanisms are expected to achieve the greatest clinical benefit. Consequently, inflammatory nociceptive pain represents the principal biological target of NSAID therapy.

### 4.2. Nociplastic Pain: When Peripheral Inflammation Is No Longer the Main Driver

Nociplastic pain is defined as pain arising from altered nociceptive processing despite the absence of clear evidence of ongoing tissue damage sufficient to activate peripheral nociceptors or a lesion of the somatosensory nervous system [34,35]. In OA, prolonged nociceptive input may induce central sensitization, resulting in amplification of pain processing that becomes increasingly independent of peripheral joint inflammation [36].

Patients with predominant nociplastic pain often present with clinical features suggestive of central sensitization, including widespread pain, hyperalgesia, allodynia, fatigue, sleep disturbances, cognitive complaints, and increased psychological distress. These manifestations frequently appear disproportionate to the extent of radiographic joint damage.

Because prostaglandin-mediated peripheral inflammation is no longer the primary driver of pain, the analgesic efficacy of NSAIDs is expected to be limited when nociplastic mechanisms predominate. In this subgroup, treatment should extend beyond anti-inflammatory therapy and incorporate interventions targeting central pain processing, including structured exercise, patient education, sleep optimization, cognitive-behavioral interventions, and centrally acting pharmacological agents when appropriate [34,35,36,37,38].

### 4.3. Neuropathic-like Pain: An Important Contributor in Selected Patients

Neuropathic pain is defined by the IASP as pain caused by a lesion or disease affecting the somatosensory nervous system [10]. It is typically characterized by burning, shooting, electric shock-like pain, paresthesia, and dysesthesia and commonly occurs in conditions such as diabetic polyneuropathy, postherpetic neuralgia, radiculopathy, spinal cord injury, or post-stroke pain [10,39,40].

Although true neuropathic pain appears to be relatively uncommon in OA, neuropathic-like symptoms are increasingly recognized in a subset of patients [41]. In many cases, these symptoms may reflect coexisting conditions such as diabetes mellitus (DM), lumbar spinal stenosis, radiculopathy, or peripheral neuropathy rather than OA itself. Nevertheless, their presence may substantially influence pain severity, physical function, and treatment response.

In a matched-pair analysis derived from the IMI Applied Public–Private Research enabling OsteoArthritis Clinical Headway (APPROACH) knee OA cohort, patients with a likely neuropathic-like pain component, defined by a PainDETECT score ≥19, exhibited greater functional impairment and more severe pain despite less severe radiographic joint damage than matched patients with PainDETECT scores ≤ 12 [42].

Because neuropathic pain mechanisms are only partially dependent on peripheral inflammation, NSAIDs alone are unlikely to provide adequate symptom control in these patients. Instead, management should include therapies directed at neural sensitization as part of an individualized multimodal treatment strategy.

Collectively, these findings indicate that OA pain is mechanistically heterogeneous and that the probability of response to NSAID therapy varies according to the dominant pain phenotype. Consequently, pain phenotyping may represent an important first step in identifying patients most likely to benefit from anti-inflammatory treatment (Table 1). Although this concept is supported by current mechanistic, translational, and clinical evidence, it should be regarded as an evidence-informed framework rather than a prospectively validated clinical decision rule.

**Figure 1:**
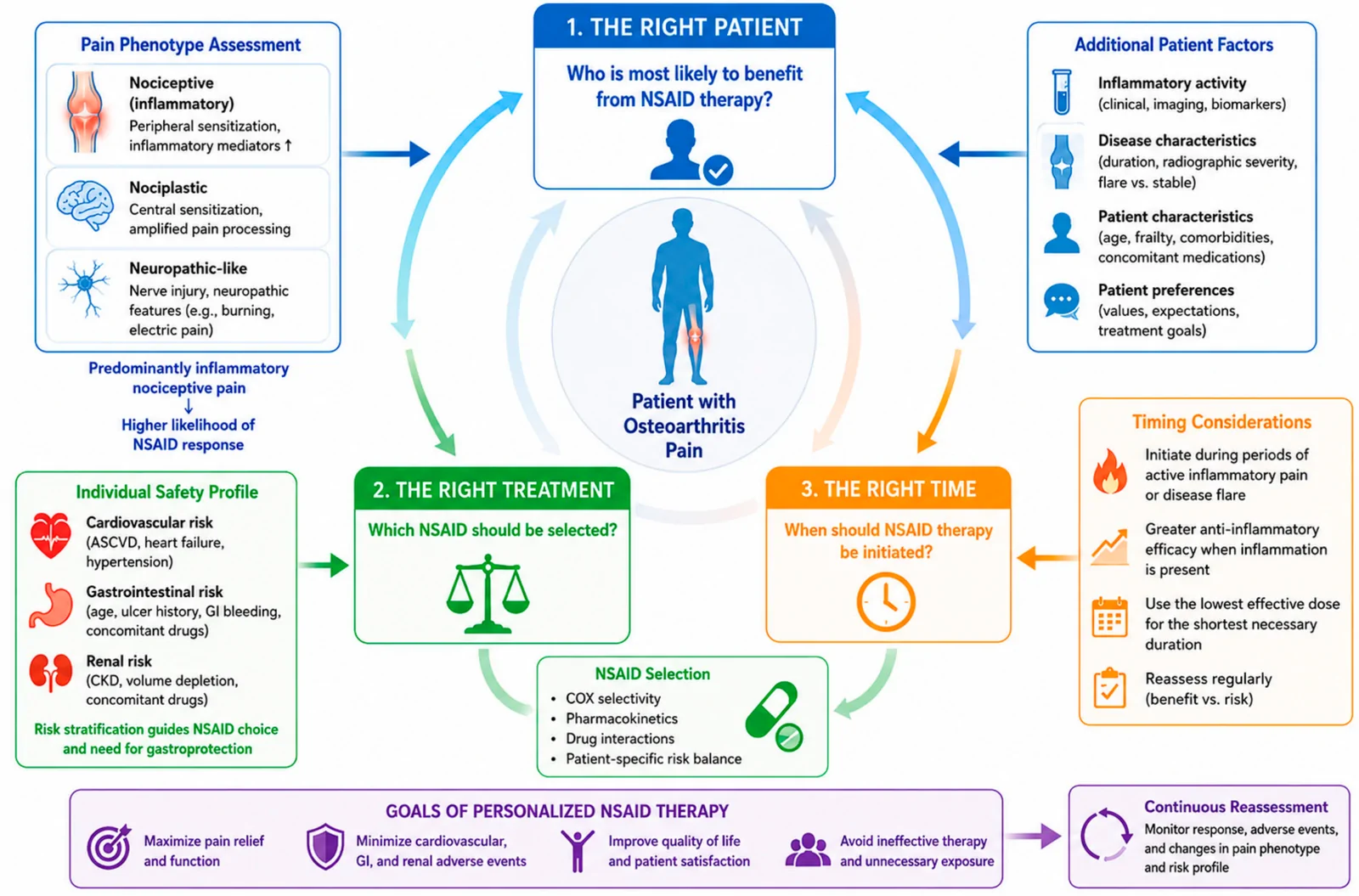
Conceptual framework illustrating the determinants of response to NSAID therapy in osteoarthritis (NSAIDs—non-steroidal anti-inflammatory drugs; ASCVD—atherosclerotic cardiovascular disease; GI—gastrointestinal; CKD—chronic kidney disease).

Taken together, these observations indicate that the variability in NSAID responsiveness is largely explained by the biological heterogeneity of OA pain. Because NSAIDs primarily target prostaglandin-mediated inflammatory nociceptive pathways, clinical benefit is expected to be greatest when inflammatory mechanisms predominate. Consequently, optimal NSAID prescribing should integrate pain phenotyping, individual cardiovascular, gastrointestinal, and renal risk assessment, and appropriate treatment timing. This evidence-informed concept forms the basis of the personalized framework proposed in this review and summarized in Figure 1.

### 4.4. Mixed Pain Phenotype and Clinical Pain Phenotyping

In routine clinical practice, OA pain rarely reflects a single pain mechanism. Many patients present with overlapping inflammatory nociceptive, nociplastic, and neuropathic-like pain mechanisms, resulting in a mixed pain phenotype. Therefore, pain phenotyping should be viewed as a dynamic clinical process aimed at identifying the predominant pain mechanism rather than assigning patients to mutually exclusive categories [34,35,43].

No single clinical finding or questionnaire is sufficiently specific to establish pain phenotype. Instead, clinicians should integrate information obtained from the medical history, physical examination, imaging findings, and validated screening instruments [34,44].

Inflammatory nociceptive pain is suggested by localized joint pain associated with synovitis, joint effusion, morning stiffness of short duration, activity-related pain, and imaging evidence of active inflammation on musculoskeletal ultrasonography or MRI, when available [45,46,47].

Neuropathic-like pain can be screened using validated questionnaires such as PainDETECT or the Douleur Neuropathique en 4 Questions (DN4). These instruments help identify neuropathic symptoms including burning pain, electric shock sensations, tingling, or dysesthesia. However, neither questionnaire establishes a definitive diagnosis, and positive results should always be interpreted in conjunction with the clinical examination [39,48,49].

Nociplastic pain may be suspected in patients presenting with widespread pain, pain disproportionate to structural joint damage, fatigue, sleep disturbances, cognitive symptoms, and generalized pain hypersensitivity. The Central Sensitization Inventory (CSI) may assist in identifying patients with central sensitization-related symptoms, although it is considered a screening questionnaire rather than a diagnostic test [44,50,51]. Where available, quantitative sensory testing (QST) may provide objective evidence of altered pain processing, including reduced pressure pain thresholds or impaired conditioned pain modulation, but its use remains largely confined to specialized clinical or research settings [44,52,53]. Taken together, these instruments should be regarded as complementary screening and supportive tools that assist clinical reasoning rather than as definitive diagnostic tests. Final pain phenotyping should always integrate clinical judgment with patient history, physical examination, imaging findings, and validated assessment tools.

Patient-reported pain distribution also provides valuable clinical information. Localized pain confined to the affected joint generally supports a predominantly nociceptive mechanism, whereas widespread or multisite pain increases the likelihood of central sensitization or mixed pain mechanisms [44,54].

Because mixed pain phenotypes are common in OA, treatment should be individualized according to the predominant pain mechanism while recognizing that multiple mechanisms may coexist. NSAIDs may remain beneficial for controlling the inflammatory nociceptive component, but patients with substantial nociplastic or neuropathic-like features frequently require multimodal management combining exercise therapy, patient education, psychological interventions when appropriate, and centrally acting or neuromodulatory medications [5,45,46].

Figure 1 summarizes the proposed evidence-informed framework for individualized NSAID prescribing in OA. The first is the patient, in whom pain phenotype and inflammatory activity determine the biological likelihood of responding to COX inhibition. The second is the drug, where differences in pharmacological properties and individual CV, GI, and renal risk tolerability profile influence the choice of the most appropriate NSAID. The third is treatment timing, emphasizing that anti-inflammatory therapy is most effective when initiated during periods of active inflammatory nociceptive pain and administered for an adequate duration. Together, these interconnected domains provide the rationale for a personalized approach to NSAID prescribing and form the basis of the clinical decision algorithm presented in the following sections.

## 5. The Right Patient: Who Should Receive NSAID Therapy?

Personalized NSAID prescribing begins with identifying the patients most likely to benefit from anti-inflammatory treatment. Although NSAIDs remain a cornerstone of pharmacological management in OA, not all patients derive the same degree of analgesic benefit. Appropriate patient selection therefore requires consideration of three key elements: the predominant pain phenotype, the presence of active inflammatory features, and the individual safety profile.

The first step is pain phenotyping. As outlined in Section 4.4, pain phenotyping should integrate clinical history, physical examination, imaging findings, and validated screening instruments rather than relying on any single clinical sign or questionnaire. Patients with predominantly inflammatory nociceptive pain represent the subgroup most likely to respond to NSAID therapy because their symptoms are primarily driven by prostaglandin-mediated peripheral sensitization. In contrast, patients in whom nociplastic or neuropathic-like mechanisms predominate are less likely to achieve meaningful pain relief from anti-inflammatory treatment alone and frequently require multimodal therapeutic approaches [1,6,25,34,42].

Once inflammatory nociceptive pain has been identified as the dominant pain mechanism, clinicians should assess whether active inflammation is contributing to symptom generation. Although OA is not traditionally regarded as a highly inflammatory disease, many patients experience episodes of synovial inflammation that contribute substantially to pain. Clinical features such as localized joint pain, tenderness on palpation, synovitis, joint effusion, morning stiffness, and intermittent inflammatory flares suggest ongoing inflammatory activity and support the use of NSAIDs [24,26,27,28,29,30,31]. When available, musculoskeletal ultrasound or MRI may provide additional evidence of synovitis and inflammatory joint activity. Although inflammatory biomarkers may offer complementary information in selected patients, imaging and biomarker assessment are not routinely recommended for guiding NSAID therapy because of limited availability, cost, and the lack of standardized implementation in routine clinical practice. From a biological perspective, these patients are expected to respond more favorably because prostaglandins (PGs), cytokines, and other inflammatory mediators play a central role in peripheral nociceptor sensitization and constitute the primary targets of COX inhibition [2,11,26,27,28,29].

The severity and functional impact of symptoms should also be incorporated into treatment decisions. Pharmacological therapy is generally most appropriate for patients experiencing clinically significant pain and impaired physical function. Validated patient-reported outcome measures, including the Visual Analog Scale (VAS), Numeric Rating Scale (NRS), and Western Ontario and McMaster Universities Osteoarthritis Index (WOMAC), provide standardized methods for assessing pain intensity, disability, and treatment response and may assist in identifying patients who are likely to benefit from NSAID therapy [2].

The anticipated analgesic benefit must always be balanced against the potential risk of adverse events. Consequently, before initiating treatment, every patient should undergo a structured assessment of CV, GI, and renal risk factors, which remain the principal determinants of NSAID safety [2,7,8,9,14,15]. Current clinical guidelines emphasize that NSAID selection should not be based solely on analgesic efficacy but should integrate individual safety profiles to minimize treatment-related complications while preserving therapeutic benefit [2,3,4,5].

Taken together, the ideal candidate for NSAID therapy is a patient with symptomatic OA who presents with predominantly inflammatory nociceptive pain, clinically meaningful pain intensity or functional limitation, evidence of active inflammatory joint involvement, and an acceptable CV, GI, and renal risk safety profile. Conversely, patients with predominant nociplastic or neuropathic-like pain mechanisms, little evidence of ongoing inflammation, or a high risk of treatment-related adverse events are less likely to benefit from NSAID therapy alone and should be considered for individualized multimodal treatment strategies. Identifying the right patient therefore represents the first and arguably the most important step toward personalized NSAID prescribing in OA.

## 6. The Right Treatment: Which NSAID Should Be Selected?

### 6.1. Are All NSAIDs Equal?

After identifying the appropriate candidate for NSAID therapy, the next step is selecting the agent that offers the most favorable balance between analgesic efficacy and safety. Although all NSAIDs share a common mechanism of action through inhibition of COX enzymes and subsequent reduction in PG synthesis, they differ substantially in their pharmacological characteristics and adverse-event profiles. Consequently, NSAIDs should not be considered interchangeable, and treatment selection should be individualized according to each patient’s CV, GI, and renal risk factors [2,7,8,9].

The pharmacological diversity of NSAIDs extends beyond their shared ability to inhibit COX enzymes. Individual agents differ in COX-1 and COX-2 selectivity, inhibitory potency, plasma half-life, tissue distribution, synovial fluid penetration, interaction with low-dose acetylsalicylic acid (ASA), and ability to cross the blood–brain barrier. These characteristics may influence dosing schedules, duration of action, tolerability, and, in selected situations, clinical effectiveness [55,56].

NSAIDs are generally classified into traditional non-selective NSAIDs, which inhibit both COX-1 and COX-2, and selective COX-2 inhibitors, which preferentially inhibit the inducible COX-2 isoenzyme. Commonly used tNSAIDs include ibuprofen, naproxen, diclofenac, indomethacin, ketoprofen, and meloxicam, whereas celecoxib and etoricoxib are the most frequently prescribed selective COX-2 inhibitors [55,56,57,58].

Importantly, greater COX-2 selectivity does not necessarily translate into superior analgesic efficacy. Clinical response depends on multiple pharmacological factors, including drug potency, administered dose, treatment duration, and pharmacokinetic properties [6,56,57,58]. For example, although diclofenac is less COX-2 selective than etoricoxib, both agents have demonstrated among the highest analgesic efficacy in network meta-analyses when administered at recommended therapeutic doses [6,7].

Consequently, NSAIDs should not be regarded as interchangeable drugs. Instead, treatment selection should consider both efficacy and safety characteristics in relation to the individual patient’s clinical profile (Table 2).

The pharmacological differences summarized in Table 2 have important clinical implications but should not be interpreted in isolation. Once an NSAID has been deemed appropriate based on pain phenotype and expected therapeutic response, the choice of a specific agent should be driven primarily by patient safety rather than by theoretical differences in analgesic efficacy. This principle is particularly relevant in OA, where most patients are older adults with multiple comorbidities and an increased susceptibility to treatment-related adverse events [2,4,5,59].

Accordingly, before initiating NSAID therapy, clinicians should perform a structured assessment of CV, GI, and renal risk factors, together with a review of concomitant medications that may increase toxicity or alter drug effectiveness [60]. Particular attention should be paid to a history of cardiovascular disease (CVD), hypertension, heart failure, previous GI ulceration or bleeding, chronic kidney disease (CKD), concomitant anticoagulant or antiplatelet therapy, glucocorticoid use, and advanced age [2,59,61,62,63,64,65]. Integrating these patient-specific factors into therapeutic decision-making allows clinicians to select the NSAID most likely to provide effective analgesia while minimizing the risk of serious adverse events.

Thus, although all NSAIDs target the same biological pathway, the optimal agent is not necessarily the most potent or the most COX-2 selective, but rather the one that best matches the individual patient’s risk profile. Personalized NSAID prescribing therefore requires balancing expected analgesic benefit with cardiovascular, gastrointestinal, and renal safety considerations [2,4,5,59].

Consequently, the benefit–risk balance of a given NSAID may vary considerably among patients despite similar levels of pain and disability, reinforcing the need for individualized treatment selection (Table 3) [2,59,63,64,65].

### 6.2. CV Risk

CV safety is a major determinant of NSAID selection in patients with OA, particularly because the disease predominantly affects older adults with a high prevalence of CV comorbidities. Although the absolute increase in CV risk associated with NSAID use is relatively small in individuals without established CVD, this risk becomes clinically significant in patients with pre-existing CVD or multiple cardiovascular risk factors [59,60].

Before initiating NSAID therapy, clinicians should perform an individualized CV risk assessment, including a history of myocardial infarction, stroke, peripheral arterial disease, heart failure, hypertension, DM, CKD, dyslipidemia, obesity, and smoking. Whenever possible, global CV risk should be estimated using validated tools such as the European Society of Cardiology SCORE2 and SCORE2-OP algorithms [61,62].

Importantly, CV safety differs among individual NSAIDs. Non-aspirin NSAIDs have been associated with an increased risk of myocardial infarction, stroke, and cardiovascular death, with excess risk becoming evident even during the first weeks of treatment [66,67]. Among traditional NSAIDs, diclofenac consistently demonstrates the least favorable CV safety profile, whereas naproxen appears to have the lowest cardiovascular risk [59,66,68].

Selective COX-2 inhibitors were introduced to reduce GI toxicity, but the withdrawal of rofecoxib highlighted concerns regarding CV safety. Nevertheless, more recent evidence, including the PRECISION trial, demonstrated that celecoxib administered at recommended doses is not associated with a higher incidence of major adverse cardiovascular events than ibuprofen or naproxen [52]. Consequently, low-dose celecoxib represents a reasonable therapeutic option in selected patients requiring NSAID therapy despite increased CV risk [52,59].

Therefore, CV risk assessment should guide NSAID selection rather than contraindicate treatment altogether. In patients with established CVD or high cardiovascular risk, naproxen or low-dose celecoxib should generally be preferred, whereas diclofenac should be avoided whenever suitable alternatives are available [59,66]. The final therapeutic decision should always balance the expected analgesic benefit against the patient’s overall cardiovascular risk profile.

### 6.3. GI Risk

GI toxicity remains one of the principal limitations of prolonged NSAID therapy. Clinical manifestations range from dyspepsia and abdominal discomfort to potentially life-threatening complications such as peptic ulcer disease, GI bleeding, perforation, and obstruction [64,65,69]. Although upper GI complications are the most widely recognized, NSAID-induced injury to the small intestine and colon is increasingly acknowledged as an important source of morbidity [63,65].

Risk factors for GI complications include previous peptic ulcer disease or GI bleeding, age over 65 years, concomitant treatment with corticosteroids, anticoagulants or antiplatelet agents, multiple NSAID use, liver cirrhosis, and severe systemic comorbidities (Table 3) [59,64,65,69]. In addition, *Helicobacter pylori* infection acts synergistically with NSAID exposure in promoting peptic ulcer disease. Screening and eradication should therefore be considered before prolonged NSAID therapy in patients at increased GI risk [59,63].

Among available NSAIDs, selective COX-2 inhibitors, particularly celecoxib, are associated with a lower incidence of upper GI complications than traditional non-selective NSAIDs. In patients at high GI risk who require anti-inflammatory therapy, current evidence supports the combination of celecoxib with a proton pump inhibitor (PPI) to maximize GI protection [59,68,69].

Accordingly, GI risk assessment should form part of every NSAID prescribing decision. Selection of the appropriate agent, use of gastroprotective therapy, and eradication of *H. pylori* when indicated allow many patients to receive effective anti-inflammatory treatment while substantially reducing the risk of serious GI complications [59,63,69].

### 6.4. Renal Risk

Renal safety represents another essential consideration when prescribing NSAIDs, particularly in older adults and patients with impaired kidney function. Although clinically significant nephrotoxicity occurs in only a minority of patients, the risk increases substantially in susceptible individuals [65,70,71].

Both traditional NSAIDs and selective COX-2 inhibitors reduce renal prostaglandin synthesis. Because PGs play a key role in maintaining renal blood flow through vasodilation of the afferent arteriole, their inhibition may decrease renal perfusion and precipitate acute kidney injury, particularly under conditions of impaired renal autoregulation [65,70]. Since COX-2 is constitutively expressed in renal tissue, selective COX-2 inhibitors do not eliminate this risk.

NSAID-related renal adverse effects include acute kidney injury, worsening of CKD, acute interstitial nephritis, sodium and water retention, edema, hypertension, hyperkalemia, and, less commonly, renal papillary necrosis [65,70,72]. Advanced age, CKD, DM, heart failure, dehydration, hypovolemia, and concomitant use of angiotensin-converting enzyme inhibitors, angiotensin receptor blockers, or diuretics markedly increase susceptibility to these complications. The combination of these three drug classes, the so-called “triple whammy”, is particularly associated with acute kidney injury [65,70,71].

Consequently, renal function should be assessed before treatment initiation and monitored during prolonged NSAID therapy in high-risk individuals [70,71]. In patients with advanced CKD, unstable renal function, or significant volume depletion, NSAIDs should generally be avoided. When treatment is unavoidable, the lowest effective dose should be prescribed for the shortest possible duration with appropriate monitoring of renal function [70,71].

### 6.5. Additional Safety Considerations

Although CV, GI, and renal complications represent the major determinants of NSAID safety, other adverse effects should also influence treatment selection.

NSAIDs may alter platelet function by inhibiting thromboxane A_2_ synthesis. Whereas aspirin produces irreversible platelet inhibition, traditional NSAIDs inhibit platelet function reversibly, with the duration of effect depending on the pharmacokinetic characteristics of each agent [56,73]. Importantly, several non-selective NSAIDs may interfere with the antiplatelet activity of low-dose aspirin, whereas selective COX-2 inhibitors generally do not [56,73]. Bleeding risk should therefore be carefully evaluated in patients receiving anticoagulants or antiplatelet therapy [59,69].

NSAID-induced hypersensitivity reactions range from mild urticaria and angioedema to severe conditions such as NSAID-exacerbated respiratory disease, anaphylaxis, DRESS syndrome, Stevens–Johnson syndrome, and toxic epidermal necrolysis [74]. Patients with asthma, chronic rhinosinusitis, nasal polyposis, or previous NSAID hypersensitivity require particular caution, and alternative analgesic strategies may be preferable in selected cases [74].

Overall, comprehensive safety assessment extends beyond CV, GI, and renal risk alone. Previous hypersensitivity reactions, bleeding risk, and concomitant medications should also be integrated into individualized therapeutic decision-making [59,69].

### 6.6. Choosing the Appropriate NSAID

No single NSAID is optimal for every patient with OA. Instead, the most appropriate agent is the one that provides the greatest likelihood of meaningful analgesia while minimizing treatment-related harm.

NSAID selection should integrate the patient’s predominant pain phenotype, evidence of active inflammation, CV, GI, and renal risk profiles, concomitant medications, previous treatment response, and patient preferences [2,59]. In patients with predominantly inflammatory nociceptive pain and a low overall risk profile, both traditional NSAIDs and selective COX-2 inhibitors represent appropriate therapeutic options. Network meta-analyses have consistently shown that diclofenac and etoricoxib provide among the greatest analgesic efficacy, while naproxen, ibuprofen, celecoxib, and meloxicam remain effective alternatives when prescribed at appropriate therapeutic doses [6,7].

In patients with increased CV risk, naproxen should generally be preferred because of its more favorable CV safety profile, whereas low-dose celecoxib may be considered in selected patients. Diclofenac should generally be avoided in patients with established CVD or high CV risk [52,59,66].

For patients at increased GI risk, selective COX-2 inhibitors, particularly celecoxib, are associated with a lower incidence of upper GI complications than traditional NSAIDs. In high-risk individuals requiring NSAID therapy, combining celecoxib with a proton pump inhibitor (PPI) provides the most favorable balance between analgesic efficacy and GI safety. Screening and eradication of *Helicobacter pylori* should also be considered before prolonged NSAID therapy in appropriate patients [59,63,68,69].

Patients with CKD, unstable renal function, dehydration, heart failure, or concomitant treatment with renin–angiotensin system inhibitors and diuretics require particular caution because of the increased risk of NSAID-induced nephrotoxicity. Whenever possible, NSAIDs should be avoided in these patients. If treatment is unavoidable, the lowest effective dose should be prescribed for the shortest possible duration with appropriate monitoring of renal function [65,70,71].

Topical NSAIDs represent an important therapeutic alternative for patients with localized OA symptoms or an increased risk of systemic adverse events, providing clinically meaningful pain relief while minimizing systemic drug exposure. Consequently, they should be considered first-line pharmacological therapy for localized OA whenever appropriate [2,4,5].

Ultimately, selecting the right NSAID requires balancing expected analgesic efficacy against individual patient safety. Integrating pain phenotype with CV, GI, renal, and patient-specific risk factors represents the cornerstone of personalized NSAID therapy in OA. The most effective NSAID is therefore not necessarily the most potent agent, but the one that offers the most favorable benefit–risk profile for the individual patient (Table 4).

## 7. The Right Time: When Should NSAID Therapy Be Initiated?

Selecting the appropriate patient and the most suitable NSAID does not, by itself, guarantee treatment success. The timing of therapy is equally important because the biological mechanisms targeted by NSAIDs evolve throughout the course of OA. As NSAIDs primarily inhibit PGs-mediated inflammatory nociceptive pathways, they are expected to provide the greatest clinical benefit when active peripheral inflammation remains a major contributor to pain generation.

Consequently, NSAID therapy should be initiated when clinical assessment suggests that inflammatory mechanisms are actively contributing to symptoms. Findings such as localized joint pain, synovitis, joint effusion, tenderness on palpation, inflammatory flares, and prolonged morning stiffness support the presence of ongoing inflammation and identify patients who are more likely to respond to anti-inflammatory treatment [24,26,27,28,29,30,31]. In contrast, pain intensity alone should not determine treatment initiation, as severe pain may also reflect central sensitization or neuropathic-like mechanisms that respond poorly to NSAIDs [1,34,35,36,37,38,39,40,41,42].

The concept of timing is particularly relevant because the relative contribution of inflammatory and central pain mechanisms may change as OA progresses. During earlier or inflammatory phases of the disease, peripheral nociceptive mechanisms often predominate and represent appropriate therapeutic targets for NSAIDs. However, persistent nociceptive input may eventually promote central sensitization, resulting in increasing contributions from nociplastic and neuropathic-like pain mechanisms. Once these mechanisms become dominant, the effectiveness of anti-inflammatory therapy is expected to decline, even when structural joint disease remains unchanged.

Appropriate treatment duration is another important component of successful NSAID therapy. Although analgesic effects may become apparent within the first few days of treatment, maximal anti-inflammatory benefit generally requires a longer therapeutic trial. Current expert recommendations suggest maintaining treatment for at least 7–10 days, with up to three weeks sometimes required to achieve the full anti-inflammatory effect [60]. Therefore, treatment failure should not be concluded after only a few days of therapy.

Conversely, prolonged NSAID administration without meaningful clinical improvement should be avoided. A recent meta-analysis demonstrated that improvements in pain and physical function generally peak within the first two weeks of treatment and gradually diminish thereafter. In contrast, GI and CV adverse events may begin to increase after approximately four weeks of continuous therapy [61]. These findings reinforce current recommendations advocating the use of the lowest effective dose for the shortest duration necessary to achieve clinically meaningful symptom control.

When an adequate therapeutic trial fails to produce significant improvement, clinicians should reconsider the underlying pain mechanism rather than simply intensifying NSAID treatment. Reassessment should include confirmation of the diagnosis, evaluation of treatment adherence, identification of predominant pain phenotype, and consideration of alternative or multimodal therapeutic strategies [60].

The timing of NSAID therapy is a critical determinant of treatment success in OA. Anti-inflammatory therapy is most effective when prescribed during periods in which inflammatory nociceptive mechanisms remain the principal drivers of pain. Delayed or prolonged treatment in patients whose symptoms are predominantly maintained by nociplastic or neuropathic-like mechanisms is unlikely to provide substantial clinical benefit, regardless of the NSAID selected [1,34,35,36,37,38,39,40,41,42].

Successful NSAID prescribing therefore requires more than choosing an effective drug. Clinicians should integrate the pain phenotype, evidence of active inflammation, the individual’s safety profile, and the appropriate treatment duration into every prescribing decision. Equally important, the response to therapy should be reassessed after an adequate trial, and treatment should be discontinued or modified if a clinically meaningful improvement is not achieved [60,61].

Ultimately, personalized NSAID therapy is based on three complementary principles: identifying the right patient, selecting the right treatment, and initiating therapy at the right time. Integrating these principles into routine clinical practice may improve pain control, optimize functional outcomes, and minimize unnecessary exposure to treatment-related adverse events.

## 8. Future Perspectives: Biomarkers, Pharmacogenetics, and Artificial Intelligence

Advances in precision medicine may further refine personalized NSAID therapy by complementing clinical pain phenotyping with molecular, imaging, pharmacogenetic, and digital biomarkers. Although current treatment decisions continue to rely primarily on clinical assessment together with CV, GI, and renal risk stratification, emerging biomarkers have the potential to improve patient selection, optimize the benefit-risk balance of NSAID therapy, and support more individualized treatment strategies [75].

Pharmacogenetic variability represents one of the most promising approaches for individualized NSAID prescribing. In particular, polymorphisms of CYP2C9, the principal enzyme responsible for the metabolism of several commonly prescribed NSAIDs, including celecoxib, diclofenac, meloxicam, and ibuprofen, may substantially influence drug exposure and toxicity. Reduced-function CYP2C9 2 and 3 alleles are associated with slower drug metabolism, increased systemic drug concentrations, and a potentially higher risk of GI, renal, and CV adverse events. Nevertheless, although genotype-guided dosing recommendations are available for selected NSAIDs, routine pharmacogenetic testing has not yet been incorporated into standard clinical practice because prospective studies demonstrating improved patient outcomes remain limited [76].

Beyond CYP2C9, numerous candidate genes have been investigated as potential determinants of NSAID responsiveness, including PTGS1, PTGS2, prostaglandin receptors, inflammatory cytokine pathways, and genes involved in nociceptive processing. While these genetic variants may contribute to interindividual differences in analgesic efficacy and adverse-event susceptibility, current evidence remains insufficient to support routine clinical implementation, and no validated pharmacogenetic profile is currently available for guiding NSAID selection in patients with OA [77,78,79].

Considerable interest has also focused on biomarkers capable of identifying inflammatory OA phenotypes. Musculoskeletal ultrasound and MRI can detect synovitis, joint effusion, and bone marrow lesions, thereby providing objective evidence of active joint inflammation that may help identify patients more likely to benefit from anti-inflammatory therapy. Likewise, circulating and synovial biomarkers reflecting cartilage metabolism, extracellular matrix turnover, and inflammatory activity have shown promise for patient stratification. However, their clinical utility remains limited by insufficient analytical standardization, inter-study variability, and the absence of validated diagnostic or therapeutic thresholds [80,81].

The identification of nociplastic pain represents another important challenge for precision medicine in OA. Biomarkers of altered central pain processing, including QST, functional MRI, and other neurophysiological assessments, have improved our understanding of central sensitization. However, these techniques remain primarily research tools because they require specialized equipment, lack standardized diagnostic criteria, and have not yet been validated for routine clinical decision-making [82,83].

Artificial intelligence (AI) and machine learning are emerging as promising tools for integrating multidimensional patient information into predictive models of treatment response. By combining clinical characteristics, imaging findings, molecular biomarkers, and pharmacogenetic information, AI-based approaches may facilitate more accurate identification of patients most likely to respond to NSAID therapy while simultaneously estimating the risk of treatment-related adverse events. Although these technologies remain investigational, they represent an important step toward precision medicine and may ultimately support personalized therapeutic decision-making in OA [84,85].

Overall, advances in pharmacogenetics, biomarker discovery, advanced imaging, and artificial intelligence are expected to substantially enhance personalized NSAID therapy. Nevertheless, robust prospective validation studies are required before these approaches can be incorporated into routine clinical practice. Until such evidence becomes available, individualized NSAID prescribing should continue to rely primarily on careful clinical pain phenotyping combined with comprehensive assessment of CV, GI, and renal safety profiles.

## 9. Clinical Decision Algorithm for Personalized NSAID Therapy

The evidence reviewed throughout this manuscript supports a shift from a diagnosis-based approach toward a mechanism-based strategy for NSAID prescribing in OA. Rather than prescribing NSAIDs solely because a patient has symptomatic OA, clinicians should first determine whether the biological mechanisms responsible for pain are likely to respond to anti-inflammatory therapy and whether treatment can be administered safely.

**Figure 2:**
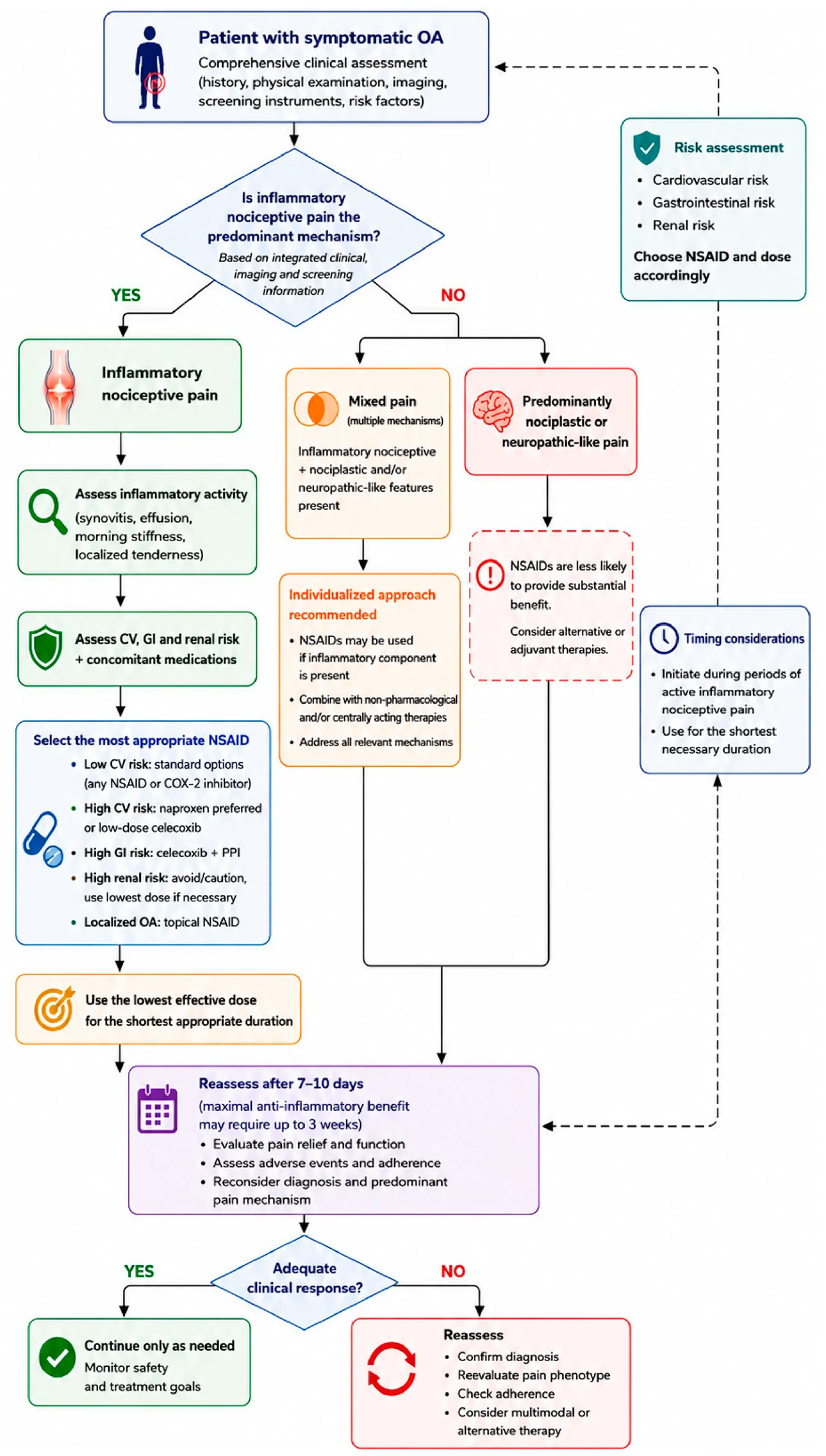
Proposed conceptual framework for phenotype-guided NSAID prescription in patients with osteoarthritis. The algorithm is intended to support clinical decision-making and reflects the current evidence together with the authors’ interpretation of mechanistic and clinical data. Prospective validation in phenotype-stratified clinical trials is warranted before routine implementation.

Based on the available evidence, we propose a practical clinical decision algorithm that integrates three sequential questions: (1) Is this the right patient? (2) What is the right treatment? (3) Is this the right time to initiate therapy? This structured approach is intended to facilitate individualized NSAID prescribing while minimizing unnecessary drug exposure and treatment-related adverse events (Figure 2).

The algorithm begins with confirmation of symptomatic OA, followed by assessment of the predominant pain phenotype. Patients with inflammatory nociceptive pain undergo evaluation of inflammatory activity and CV, GI, and renal risk factors before selection of the most appropriate NSAID. Treatment is initiated using the lowest effective dose for the shortest appropriate duration, followed by reassessment of efficacy and safety after an adequate therapeutic trial. Patients demonstrating insufficient clinical response should undergo reassessment of pain phenotype and consideration of alternative or multimodal therapeutic approaches.

The conceptual framework proposed in this review integrates different levels of evidence. Some components are supported by RCTs and meta-analyses (e.g., NSAID efficacy and safety), whereas others are informed primarily by observational studies, mechanistic and translational research, or the authors’ synthesis of the available literature. Accordingly, the proposed framework should be interpreted as an evidence-informed conceptual model intended to support clinical reasoning rather than as a validated clinical decision rule. Prospective studies stratifying patients according to pain phenotype are needed to validate this approach.

The first step is to identify patients with symptomatic OA whose pain is predominantly driven by inflammatory nociceptive mechanisms. Clinical assessment should include pain phenotype, evidence of active inflammation, symptom severity, and functional impairment. Patients presenting with localized inflammatory pain, synovitis, joint effusion, tenderness, or inflammatory flares are the most appropriate candidates for NSAID therapy. In contrast, patients with predominant nociplastic or neuropathic-like pain should undergo further evaluation and consideration of alternative or multimodal therapeutic strategies before escalation of anti-inflammatory treatment.

Once an appropriate candidate has been identified, the second step is selection of the NSAID according to the patient’s individual safety profile. CV, GI, and renal risk assessment should precede every prescription. Patients with elevated CV risk may benefit from naproxen or low-dose celecoxib, whereas diclofenac should generally be avoided in established CVD. Patients at increased GI risk should preferentially receive celecoxib combined with proton pump inhibitor therapy, while individuals with impaired renal function require cautious use, dose limitation, or avoidance of NSAIDs depending on the severity of renal dysfunction. Topical NSAIDs should be considered whenever symptoms are localized, and systemic adverse effects are a concern.

The third step is determining the optimal timing and duration of therapy. NSAIDs should be initiated when inflammatory nociceptive mechanisms are clinically active and should be continued only for the period necessary to achieve meaningful symptom control. Treatment response should be reassessed after an adequate therapeutic trial, typically 7–10 days, recognizing that maximal anti-inflammatory benefit may require up to three weeks. If clinically relevant improvement is not achieved, clinicians should reconsider the diagnosis, reassess the dominant pain phenotype, evaluate treatment adherence, and modify the therapeutic strategy rather than simply increasing NSAID exposure.

This algorithm emphasizes that successful NSAID prescribing is not determined by the choice of drug alone but by integrating pain biology with individualized risk assessment and appropriate treatment timing. Such an approach is consistent with the principles of precision medicine and provides a practical framework for translating current evidence into everyday clinical practice.

## 10. Limitations and Future Perspectives

This narrative review has several limitations. First, unlike a systematic review or meta-analysis, the literature selection was not based on a predefined protocol or formal assessment of methodological quality. Consequently, although the review integrates evidence from rRCTs, observational studies, clinical guidelines, and mechanistic research, the conclusions should be interpreted as a synthesis of the current body of evidence rather than a quantitative evaluation of treatment effects.

Second, although increasing evidence supports the concept that pain phenotype influences responsiveness to NSAID therapy, high-quality prospective studies specifically evaluating phenotype-guided NSAID treatment remain limited. Most available data are indirect and derive from studies investigating pain mechanisms rather than randomized trials stratified according to nociceptive, nociplastic, or neuropathic-like pain phenotypes.

Third, pain phenotyping itself remains challenging in routine clinical practice. Although validated questionnaires and clinical assessment tools are increasingly available, no universally accepted gold standard currently exists for classifying pain phenotypes in patients with OA. Furthermore, individual patients frequently exhibit overlapping pain mechanisms that evolve during disease progression, making strict categorization difficult.

Another important limitation is that safety considerations largely guide current NSAID prescribing, whereas reliable biomarkers capable of predicting therapeutic response are still lacking. Future advances in imaging, molecular biomarkers, digital phenotyping, and artificial intelligence may improve the identification of patients who are most likely to benefit from anti-inflammatory therapy and facilitate implementation of precision medicine in OA.

An important limitation of the proposed framework is that it has not yet been prospectively validated in RCTs specifically stratifying patients according to pain phenotype. Consequently, the proposed algorithm should be considered hypothesis-generating and intended to facilitate clinical reasoning rather than replace individualized clinical judgment.

Most of the evidence discussed in this review originates from studies of knee OA, which represents the most extensively investigated OA phenotype. Although the proposed personalized framework is based on biological mechanisms that are likely to be relevant across different OA sites, caution is warranted when extrapolating these recommendations to hip, hand, or other joint locations, where pain mechanisms, clinical presentation, and the available evidence may differ. Future joint-specific studies are needed to validate this framework across the spectrum of OA.

Despite these limitations, the proposed framework provides a clinically relevant approach that integrates current knowledge regarding pain mechanisms with established CV, GI, and renal safety assessment. Rather than replacing existing guideline recommendations, this framework is intended to complement them by supporting more individualized clinical decision-making. Future prospective studies are needed to validate phenotype-guided NSAID prescribing algorithms and to determine whether personalized treatment strategies improve pain control, functional outcomes, and long-term safety compared with conventional approaches.

## 11. Conclusions

The management of OA pain is evolving from a traditional diagnosis-based approach toward a more individualized understanding of pain mechanisms. Rather than representing a uniform consequence of structural joint degeneration, OA pain is now recognized as a heterogeneous and dynamic condition in which inflammatory nociceptive, nociplastic, and neuropathic-like mechanisms contribute to symptom generation to varying degrees. This biological heterogeneity largely explains why patients receiving the same NSAID under similar treatment conditions may experience markedly different clinical outcomes.

Although NSAIDs remain the cornerstone of pharmacological treatment for OA, their effectiveness depends on more than the intrinsic properties of the selected drug. Optimal outcomes require careful identification of patients whose pain is predominantly driven by inflammatory nociceptive mechanisms, thoughtful selection of the most appropriate NSAID according to individual CV, GI, and renal risk profiles, and initiation of therapy when inflammation remains an important contributor to pain. Personalized NSAID prescribing therefore extends beyond improving safety; it also aims to maximize therapeutic response while avoiding unnecessary treatment in patients unlikely to benefit.

The framework proposed in this review integrates these principles into an evidence-informed conceptual approach intended to support individualized clinical decision-making rather than replace current guideline-based management. Although current mechanistic, translational, and clinical evidence supports this strategy, prospective studies are required to validate phenotype-guided NSAID prescribing in routine clinical practice.

Future research should focus on validating phenotype-guided treatment algorithms, identifying reliable biomarkers of NSAID responsiveness, and integrating clinical, imaging, molecular, and pharmacogenetic data to refine patient stratification. As evidence continues to evolve, personalized NSAID therapy may become an increasingly important component of precision medicine in osteoarthritis.

Ultimately, optimizing NSAID therapy in OA is likely to depend not only on selecting an effective drug, but also on identifying the right patient, choosing the right treatment, and initiating therapy at the right time.

## Figures and Tables

**Table 1:** Clinical characteristics of OA pain phenotypes and expected response to NSAIDs.

Pain Phenotype	Main Mechanism	Clinical Characteristics	Expected Likelihood of Clinical Benefit from NSAID Therapy *	Clinical Implication
Nociceptive inflammatory	Peripheral sensitization, prostaglandins, cytokines	Localized pain, synovitis, effusion, morning stiffness	More likely to benefit	NSAIDs should be considered when clinically indicated
Mixed pain	Combined peripheral and central sensitization	Persistent pain with intermittent inflammatory flares	May benefit in selected patients	NSAIDs may be considered as part of a multimodal treatment strategy
Neuropathic-like	Neural dysfunction and abnormal pain signaling	Burning pain, electric shocks, dysesthesia	Less likely to benefit	Consider assessment for neuropathic pain and neuromodulatory therapies
Nociplastic	Central sensitization	Widespread pain, hyperalgesia, sleep disturbance, fatigue	Unlikely to derive substantial benefit	Prioritize non-pharmacological interventions and centrally acting therapies when appropriate

* The estimated likelihood of NSAID responsiveness is based on the integration of current mechanistic knowledge, clinical pharmacology, observational evidence, and available randomized clinical trials. These categories represent the authors’ conceptual interpretation of the current evidence and have not yet been prospectively validated in randomized trials stratifying patients according to pain phenotype.

**Table 2:** Commonly used NSAIDs in OA and typical effective doses (NSAIDs—non-steroidal anti-inflammatory drug; COX-2—cyclooxygenase; CV—cardiovascular; GI—gastrointestinal).

NSAID	Class	Typical OA Dose	Comments
Ibuprofen	Non-selective NSAID	1200–2400 mg/day	Lower CV risk, shorter half-life
Naproxen	Non-selective NSAID	500–1000 mg/day	Generally considered to have the most favorable CV safety profile among traditional NSAIDs
Diclofenac	Traditional NSAID with relative COX-2 preference	100–150 mg/day	Among the most effective NSAIDs in network meta-analyses at therapeutic doses
Celecoxib	Selective COX-2 inhibitor	200 mg/day (maximum 400 mg/day)	Lower risk of upper GI complications than non-selective NSAIDs
Etoricoxib	Selective COX-2 inhibitor	30–60 mg/day	Among the most effective NSAIDs; use with caution in patients at increased CV risk
Meloxicam	Preferential COX-2 inhibitor	7.5–15 mg/day	Intermediate profile
Ketoprofen	Non-selective NSAID	100–200 mg/day	Widely used in Europe
Topical diclofenac	Topical NSAID	According to the formulation	Reduced systemic exposure

**Table 3:** Major CV, GI, and renal risk factors to consider before initiating NSAID therapy in patients with OA (NSAIDs—non-steroidal anti-inflammatory drug, CV—cardiovascular, GI—gastrointestinal, CKD—chronic kidney disease, DM—diabetes mellitus).

Risk Factors		
CV	GI	Renal
Previous myocardial infarction	Previous complicated ulcer	CKD
Previous stroke	Previous GI bleeding	Acute kidney injury
Peripheral arterial disease	Age >65 years	Older age
Heart failure	Multiple NSAID use	Dehydration
Hypertension	Concomitant corticosteroids	DM
DM	Anticoagulants/antiplatelets	Hypertension
Hyperlipidemia	Aspirin use	Angiotensin-Converting Enzyme inhibitors
Smoking	Clopidogrel/ticlopidine	Angiotensin receptor blockers
Obesity	Dyspepsia history	High-dose diuretics
Established CV disease	Liver cirrhosis	Nephrotoxic medications
Concomitant antiplatelet therapy	Hospitalization history	Frequent contrast-media exposure

**Table 4:** Practical selection of NSAIDs according to patient risk profile (CV—cardiovascular; GI—gastrointestinal; NSAIDs—nonsteroidal anti-inflammatory drugs; PPI—proton pump inhibitor).

Clinical Profile	Preferred NSAID Strategy
**Low CV/Low GI risk**	Any appropriate NSAID according to individual patient characteristics (e.g., diclofenac, naproxen, celecoxib, etoricoxib)
**High CV/Low GI risk**	Naproxen preferred; low-dose celecoxib may be considered in selected patients
**Low CV/High GI risk**	Celecoxib + PPI
**High CV/High GI risk**	Avoid NSAIDs whenever possible; if treatment is necessary, carefully individualize therapy and consider gastroprotection
**High renal risk (CKD or unstable renal function)**	Avoid NSAIDs whenever possible; if unavoidable, use the lowest effective dose for the shortest duration with renal monitoring
**Localized OA**	Topical NSAIDs should be considered as first-line therapy

The recommendations presented in this table represent a simplified clinical framework based on current international guidelines and should always be interpreted within an individualized benefit–risk assessment.

## Data Availability

No new data were created or analyzed in this study. Data sharing does not apply to this article.

## Abbreviations

ASA	Low-dose acetylsalicylic acid
ASCD	Atherosclerotic cardiovascular diseas
CKD	Chronic kidney disease
CNS	Central nervous system
COX	Cyclooxygenase
CV	Cardiovascular
CVD	Cardiovascular disease
DM	Diabetes mellitus
GI	Gastrointestinal
IASP	International Association for the Study of Pain
NGF	Nerve growth factor
NSAIDs	Non-steroidal anti-inflammatory drugs
PG	Prostaglandin
PPI	Proton pump inhibitor
RCT	Randomized controlled trials
